# Multidisciplinary Approach to Patient-Specific Implants (PSIs): A Case Report and Review of Literature

**DOI:** 10.7759/cureus.41238

**Published:** 2023-06-30

**Authors:** Vedha Vivigdha, Murugesan Krishnan, Santhosh Kumar M P, Senthil Murugan P, Preethi Rajamanickam

**Affiliations:** 1 Oral and Maxillofacial Surgery, Saveetha Institute of Medical and Technical Sciences, Chennai, IND; 2 Orthodontics and Dentofacial Orthopaedics, Saveetha Institute of Medical and Technical Sciences, Chennai, IND

**Keywords:** multidisciplinary decision-making, conventional orthognathic surgery, 3d surgical navigation, cad-cam milling, anchorage, skeletal anchorage, temporary anchorage device, customised implants, patient specific implants-psi, patient specific implants

## Abstract

Orthodontic treatment often faces challenges in achieving proper anchorage. While orthodontic mini-implants have gained popularity, no universally accepted design and insertion protocol exists for these implants. However, their relatively modest failure rate indicates their clinical reliability. To address complex geometries in the maxilla and mandible, patient-specific implants (PSIs) have emerged as a solution. PSI is currently employed in various domains of oral and maxillofacial surgery like temporomandibular joint (TMJ), total joint replacement, reconstruction of the facial skeleton, and orthognathic surgery. PSI allows for the creation of customized implant fits, leading to shorter rehabilitation times. This case report presents a multidisciplinary approach involving oral surgery and orthodontics, specifically focusing on the design of PSI, surgical placement of PSI, and use of PSI in maxillary protraction in orthodontics. The report highlights the design process of designing PSI and emphasizes its role in orthodontic treatment. By incorporating PSI as a temporary anchorage device (TAD), enhanced stability, precise control over tooth movement, and accurate repositioning of jaws can be achieved. The collaborative effort between orthodontists and oral surgeons is crucial in integrating PSI into the overall treatment plan. Despite the higher costs associated with PSI, their numerous advantages outweigh these drawbacks. PSI plays a vital role in providing enhanced stability, appropriate treatment plan, and achieving desired treatment in orthodontic and oral surgery procedures.

## Introduction

Orthodontists face significant challenges when it comes to achieving proper anchorage during orthodontic treatment. To address this issue, orthodontic mini-implants have become widely utilized, despite the absence of a universally accepted design and insertion protocol. These mini-implants have shown a relatively modest reported failure rate of 13.5%, indicating their clinical reliability as orthodontic devices [1]. The introduction of mini-implants as temporary anchorage devices (TADs) has not only expanded the possibilities for tooth movement without the use of headgear but has also influenced the management of various orofacial deformities, malocclusions, and space problems prior to prosthetic replacement of missing teeth [2]. In recent years, mini-screws have emerged as an alternative to highly invasive techniques, serving as TADs for a range of orthodontic tooth movements, including forced eruption. Primary stability is considered a critical factor in assessing the success rate of these orthodontic devices.

Patient-specific implants have been introduced to address complex geometries found in the maxilla and mandible. PSI allows for the creation of customized implant that fits quickly and with greater accuracy, leading to shorter rehabilitation times and overall cost reduction. This technique involves extensive preoperative planning based on CT or MRI images, following the manufacturer's guidelines, and employing specialized software programs to manufacture disposable cutting blocks tailored to each patient's unique needs. Recent advancements in computer-aided design and computer-aided manufacturing (CAD/CAM) technology have facilitated the application of personalized medicine in oral and maxillofacial surgery, leading to improved outcomes. These developments have allowed for greater precision and customization in treatment planning and implementation. Furthermore, the decreasing cost of this technology has made it more affordable and accessible to patients [3]. In this case report, we present a multidisciplinary approach involving oral surgery and orthodontics which highlights our experience with PSI and their role in maxillary protraction in orthodontics. The report emphasizes the design process involved in creating PSI and the significant impact that PSI has had on orthodontic treatment.

## Case presentation

**Figure 1:**
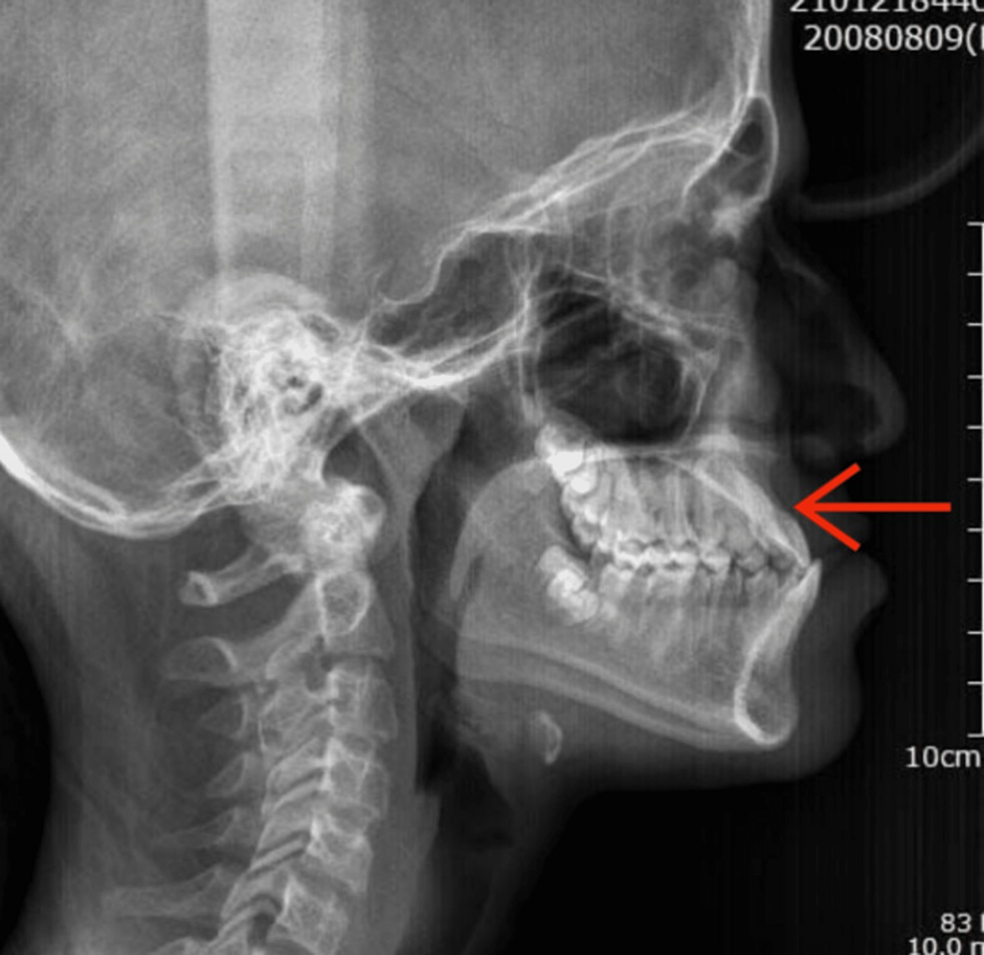
Preoperative radiograph. Depicts the preoperative radiograph showing Class III malocclusion. The arrow mark depicts the retrognathic maxilla in the lateral cephalogram.

A 13-year-old female patient presented with a chief complaint of forwardly placed lower teeth. On examination, she had a concave profile and an anteriorly divergent face, and the patient had skeletal class III malocclusion attributable to a retrognathic upper jaw (Figure 1). To address this issue, bone-anchored maxillary protraction is planned with PSI.

Designing of patient-specific implant

**Figure 2:**
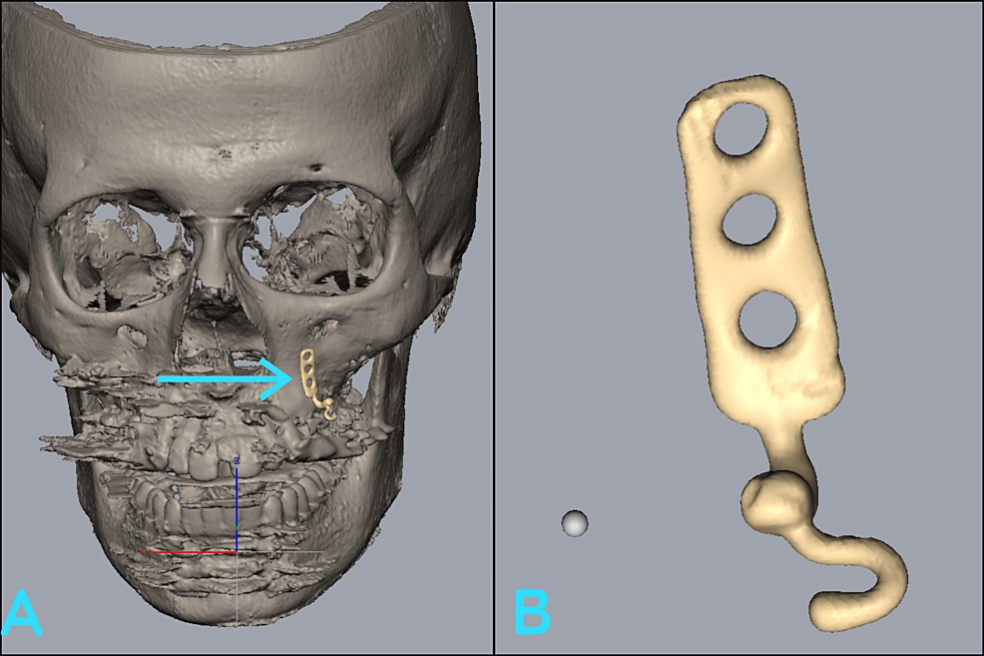
Design of patient-specific implant. Represents the stereolithographic model showing patient-specific implant (A) at the upper left quadrant and design of patient-specific implant (B) using Geomagic Software.

The planning and manufacturing process of PSI involves several steps. Initially, cone-beam computed tomography (CBCT) was taken and the data from a CBCT scan was obtained in a DICOM file format. This was then transferred to Geomagic Software (3D Systems, NC, USA) for the fabrication of the patient-specific plates. This DICOM file is then converted into a stereolithographic (STL) format, which accurately represents the dimensions of the implant. The STL file is further converted into an SLI file format, which structures the implant into layered components, typically with a thickness of around 30 microns (Figure 2). The SLI file is subsequently sent to a 3D printer, where the implants were then three-dimensionally printed using selective laser melting with a 1.5-mm thickness of titanium metal using additive manufacturing techniques that create the implant layer by layer. Once the printing process is completed, the implant is removed from the printing platform and its supports. It undergoes polishing, wiping, and sterilization procedures to ensure cleanliness and sterility. Finally, the implant is packaged and delivered to the surgical team. Throughout this process, the orthodontist and surgeons helped design and approve the implant. Their expertise and involvement are essential in ensuring that the implant meets the specific requirements of the patient and the surgical procedure.

Surgical procedure

**Figure 3:**
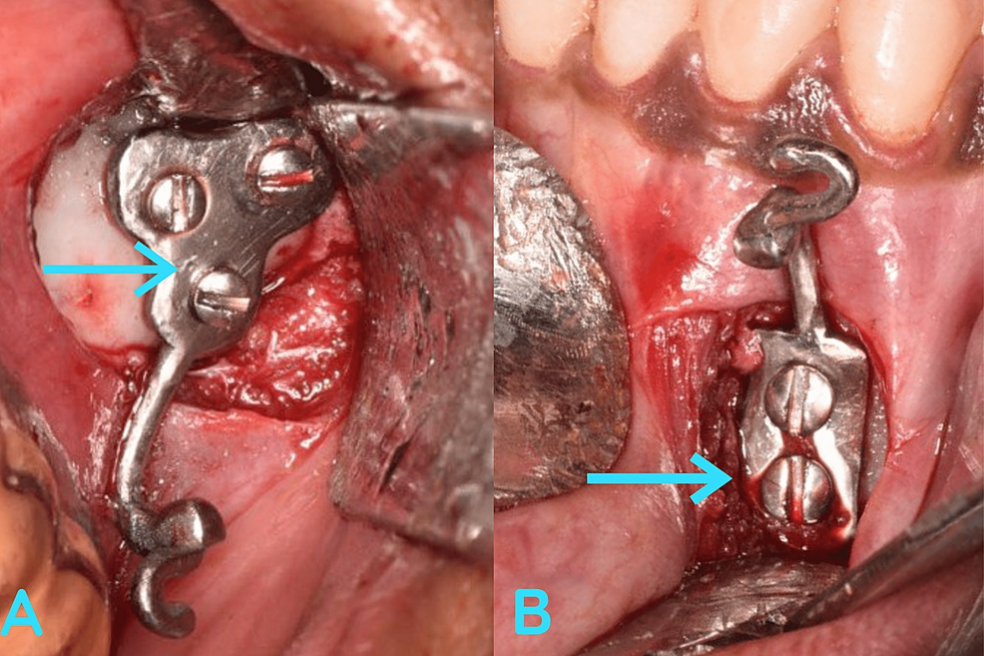
Intraoperative image. Intraoperative image showing the placement of PSI. The arrow mark depicts the PSI placed in the maxilla (A) and mandible (B) PSI, patient-specific Implant

**Figure 4:**
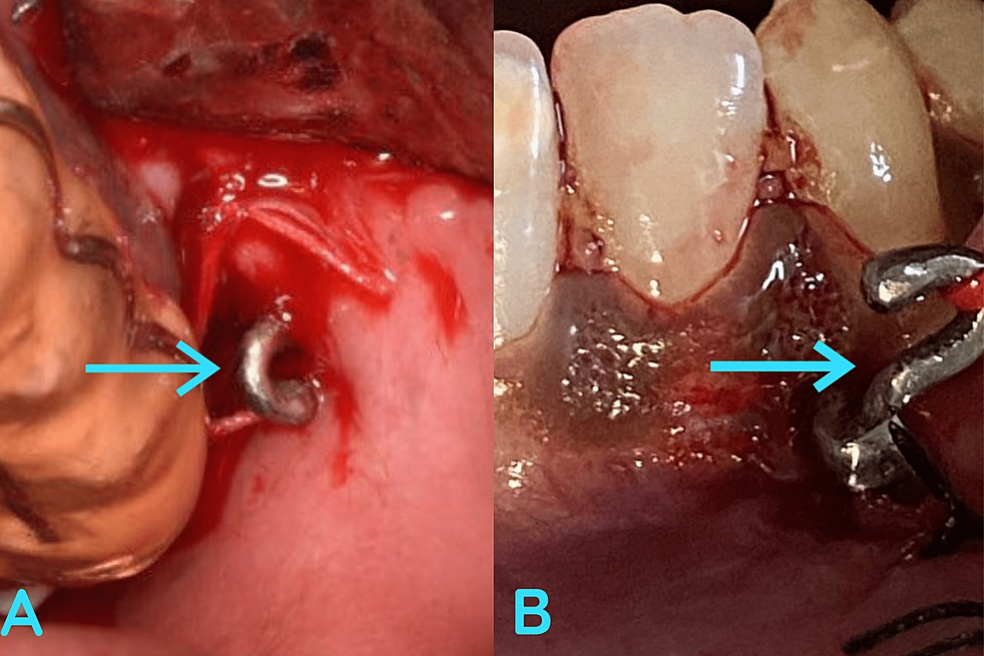
Postoperative image. Represents the intra-oral closure done using 3.0 polyglactin. Note the arrow mark represents the hook of the PSI projecting intraorally for the attachment of elastics. PSI, patient-specific implant

**Figure 5:**
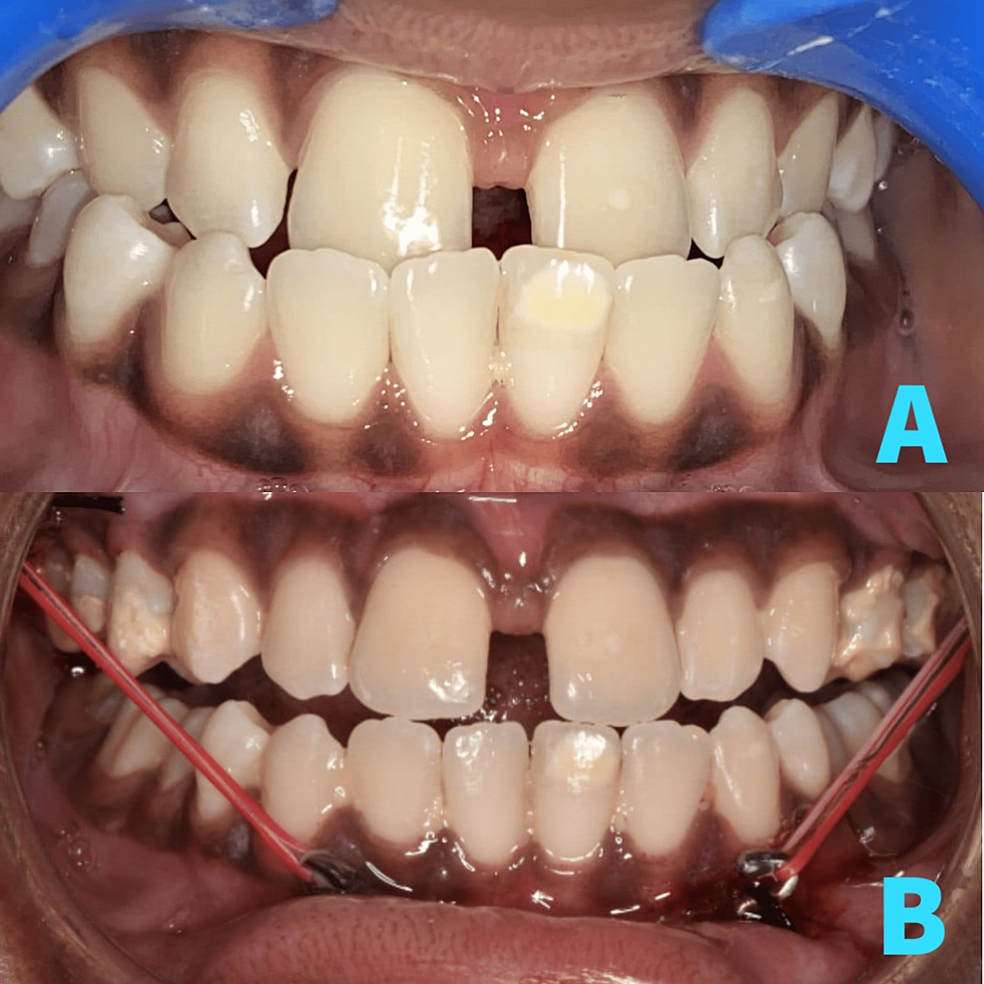
Pre- and postoperative image. Pre-operative occlusion before placement of PSI (A). Post-operative occlusion showing attachment of elastics to PSI (B). PSI, patient-specific implant

Under local anesthesia, a vestibular incision was placed in relation to the upper and lower premolars, and following that the mucoperiosteal flap was reflected. PSI were placed and secured with three titanium screws on each implant (Figure 3). The closure was done with 3.0 polyglactin (3.0 Vicryl) (Figure 4). The hooks were exposed intraorally, where the elastics were placed postoperatively to achieve the desired orthodontic movement (Figure 5).

 

## Discussion

Orthodontic implants are utilized for defined durations and many of which are not endosseous dental implants. In literature, alternative terminologies have been employed like miniscrew implants, microscrew implants, and TADs [4]. The concept of utilizing metallic screws as anchorage devices were proposed by Gainsforth and Higley as early as 1945 [5]. Creekmore and Eklund were the pioneers in introducing screws for the exclusive purpose of orthodontic anchorage, marking a significant milestone in clinical orthodontics in 1983 [6]. Over the past two decades, the utilization of PSI has significantly increased due to advancements in three-dimensional (3D) CAD and CAM technologies across various medical fields. Notable applications of PSI can be observed in orthopedic surgery, specifically in hip and knee arthroplasty, and cranial surgery [5]. Moreover, PSI has found use in oral and maxillofacial surgery for various purposes, including reconstruction of orbital defects, facial contouring, mandibular reconstruction, dental rehabilitation, temporomandibular joint (TMJ) prosthesis, and orthognathic surgery [6]. Various PSI designs have been used in orthognathic surgeries like Le Fort I osteotomy, bilateral sagittal split osteotomy [7], and genioplasty.

The use of PSI for orthodontic anchorage is still a new technique. While orthodontic anchorage has been understood since the 17th century, it was not explicitly defined until 1923 by Louis Ottofy [8] as "the base against which orthodontic force or reaction of orthodontic force is applied.” TADs are devices temporarily fixed to the bone to enhance orthodontic anchorage. They can support the teeth of the reactive unit or eliminate the need for a reactive unit. TADs can be located transosteally, subperiosteally, or endosteally, and can be mechanically or biochemically fixed to bone. It is important to note that dental implants used alone for supporting prostheses are not considered TADs as they are not removed after orthodontic treatment [9-10]. The incorporation of dental implants and TADs in orthodontics allows for infinite anchorage, characterized by zero anchorage loss due to reaction forces [11-12].

Computer-designed PSI provides several advantages, including increased accuracy, improved adaptation to defects, enhanced stability, predictable outcomes, and superior refinement of facial contours [13]. The multidisciplinary approach involves close collaboration between Orthodontists and Oral surgeons to ensure the proper integration of PSI into the overall treatment plan. From our perspective, the utilization of PSI does present a notable drawback in terms of the higher cost involved, which may influence patients to explore more economical options. However, we firmly maintain that the multitude of advantages offered by PSIs surpasses this disadvantage.

## Conclusions

Patient-specific implants are crucial in enhancing stability during orthodontic treatment or oral surgery procedures. The treatment algorithm presented here provides the desired anchorage thereby achieving maxillary protraction without a time-consuming treatment protocol. By incorporating PSI as TADs, enhanced stability, precise control over tooth movement, and accurate repositioning of jaws can be achieved. This collaborative effort helps to optimize the appropriate treatment plan, success, and outcomes of orthodontic and oral surgery procedures.
